# Investigation of the role of multidrug resistance proteins (MRPs) in vascular homeostasis

**DOI:** 10.1186/2050-6511-16-S1-A33

**Published:** 2015-09-02

**Authors:** Robert M H Allen, Aniruthan Renukanthan, Kristen J Bubb, Inmaculada C Villar, Amie J Moyes, Reshma S Baliga, Adrian J Hobbs

**Affiliations:** 1 William Harvey Research Institute, Barts & The London Medical School, Queen Mary University of London, London, UK

## Background

Cellular levels of cyclic GMP (cGMP) are tightly controlled by synthetic and degradative mechanisms. Pharmacological manipulation of these processes (e.g. soluble guanylate cyclase stimulators, phosphodiesterase 5 inhibitors) augments cGMP-dependent signalling and is beneficial in treating cardiovascular disease (e.g. pulmonary hypertension). An additional mechanism potentially important in regulating cGMP signalling is cellular extrusion, driven by a family of multidrug resistance proteins (MRPs). Herein, we investigated if inhibition of MRPs modulates vascular reactivity, smooth muscle cell proliferation, and systemic hemodynamics.

## Methods and main findings

The functional reactivity of murine aortic rings and proliferation of human pulmonary artery smooth muscle cells (PASMC) were determined in the absence and presence of the MRP inhibitor MK571. Hemodynamic changes in vivo in response to MK571 were analysed acutely by bolus dosing and chronically by radiotelemetry.

MK571 (1nM-50µM) caused a concentration-dependent relaxation of mouse aortic rings. In the presence of a threshold concentration of MK571 (3µM), vasorelaxant responses to NO and atrial natriuretic peptide (ANP) were significantly augmented. MK571 (3µM) also significantly inhibited PASMC proliferation and enhanced the anti-mitogenic properties of ANP (1µM) and NO. *In vivo*, MK571 (0.001-10mg/kg; i.v.) elicited an acute, dose-dependent hypotensive activity and when delivered via the drinking water caused a more sustained drop in mean arterial pressure (~5 mmHg).

## Conclusion

These data suggest that extrusion by MRPs contributes to the dynamic equilibrium regulating intracellular levels of cGMP, and may represent a further target amenable to drug intervention for the treatment of cardiovascular disease.

